# Metastatic Renal Cell Carcinoma to the Testis: A Clinicopathologic Analysis of Five Cases

**DOI:** 10.1155/2020/9394680

**Published:** 2020-03-02

**Authors:** Gang Wang, Chen Zhou, Carlos F. Villamil, Alan So, Ren Yuan, John C. English, Edward C. Jones

**Affiliations:** ^1^Department of Pathology, BC Cancer Vancouver Centre, Vancouver, BC, Canada; ^2^Department of Urology, Vancouver General Hospital, Vancouver, BC, Canada; ^3^Department of Radiology, BC Cancer Vancouver Centre, Vancouver, BC, Canada; ^4^Department of Pathology, Vancouver General Hospital, Vancouver, BC, Canada

## Abstract

The testicular spread of renal cell carcinoma is extremely rare. Five cases of renal cell carcinoma metastatic to the testis are described. The patients ranged from 45 to 81 years of age. Four of the five patients had known renal cell carcinoma. The time intervals between the partial and radical nephrectomies for the primary kidney tumors and the occurrence of testicular metastases ranged from 29 to 34 months. In one patient, the testicular mass was the initial presentation leading to a diagnosis of renal cell carcinoma. There were three ipsilateral metastases, one contralateral metastasis, and one bilateral metastasis. The metastatic deposits ranged in size from 2.0 to 5.7 cm. One case had multiple metastatic tumor nodules. All of the metastatic tumors had clear cell histological features, microscopically concordant with the primary renal cell carcinoma subtype. Three patients died of the disease 17 to 42 months after orchiectomy. One patient is alive with additional metastatic lesions 13 months after orchiectomy. One patient had been free of disease at 87 months after orchiectomy but is now on targeted therapy for an additional metastasis at 93 months after orchiectomy. To date, this report is one of the largest single series of patients with renal cell carcinoma metastatic to the testis, and it has the longest follow-up and survival among all the reported cases.

## 1. Introduction

Secondary involvement of the testis by metastatic carcinoma is rare. Most often, it is an incidental autopsy finding. However, occasionally, a tumor metastatic to the testis may be the initial presentation [1]. In this circumstance, it could be misdiagnosed as one of the many types of primary testicular tumors. Most reported examples of metastatic carcinoma in the testis have been individual case reports [1–19]. Metastatic renal cell carcinoma (RCC) involving the testis comprises a small portion of this group of cases. One small case series of metastatic renal cell carcinoma to the testis has been previously reported [5]. We report an additional five cases of RCC metastatic to the testis and review the literature on this rare clinical manifestation. Two of the cases were the subject of a prior review by our own group [20] but are included in this series so that this report represents an overall consideration of our entire experience with this rare clinical scenario.

## 2. Materials and Methods

The 5 cases were all obtained from the BC Cancer Agency tumor registry for the period of 1987 to 2017. Data including initial presentation, gross findings, and follow-up information were collected from the clinical records. All histology slides from each case were reviewed to confirm the diagnosis and additional pathology findings. The clinical information is summarized in Table 1.

## 3. Clinical Features

### 3.1. Case 1

A 53-year-old man, with known Von Hippel-Lindau syndrome, underwent radiofrequency ablation twice for left kidney clear cell RCC, WHO/ISUP grade 1. He subsequently underwent a left partial nephrectomy for locally recurrent or persistent tumor of the same type and grade. He also had a Whipple's pancreatectomy for five separate pancreatic neuroendocrine tumors. He was found to have right testicular enlargement 33 months after his partial nephrectomy. Ultrasound showed bilateral diffuse abnormal testicles. A diagnostic right radical orchiectomy was performed, followed by a left radical orchiectomy three months later. Pathologic examination of both testes demonstrated bilateral metastatic low-grade clear cell RCC. Magnetic resonance imaging of the abdomen demonstrated multiple tumors in both kidneys consistent with RCC, with the largest tumor measuring 2.6 cm in size. The largest tumor was treated by radiofrequency ablation. The smaller tumors have been followed radiographically and have remained stable. Postorchiectomy surveillance for 24 months has revealed no evidence of additional metastatic disease.

### 3.2. Case 2

An 81-year-old man had a history of clear cell RCC, WHO/ISUP grade 3, TNM stage pT2a, of the left kidney. This was treated with a left radical nephrectomy. A mass was found in his left testis 34 months after the nephrectomy. A radical orchiectomy was performed. Pathologic examination demonstrated a metastatic clear cell RCC to the testis. The patient was found to have bony metastases in the right humerus and left tibia and in the left maxillary sinus at 17 months and 31 months postorchiectomy, respectively. He succumbed to metastatic disease 34 months following the orchiectomy.

### 3.3. Case 3

A 45-year-old man was diagnosed with RCC of the right kidney. He underwent a right radical nephrectomy. Pathologic examination revealed a clear cell RCC, WHO/ISUP grade 4, 12 cm in size, with vascular invasion, TNM stage pT3a. He was found to have tumors in his right testis and left lung 38 months postnephrectomy, confirmed to be metastatic high-grade RCC upon radical orchiectomy and lung biopsy. In spite of treatment with chemotherapy, an additional metastasis to the vertebral spine was found 39 months postnephrectomy. Radiotherapy was given. He died of metastatic disease three months later.

### 3.4. Case 4

A 63-year-old man presented with a right testicular mass. Further investigation demonstrated multiple metastatic tumors to the brain, liver, lung, bone, and duodenum. Two tumors were identified in the right kidney. Radical orchiectomy with pathologic examination revealed a metastatic high-grade RCC with sarcomatoid dedifferentiation. The patient was treated with systemic therapy of vascular endothelial growth factor (VEGF) inhibitor and external beam radiation therapy to the brain. He died of metastatic disease seventeen months following the orchiectomy.

### 3.5. Case 5

A 76-year-old man had an incidental 4 cm left renal mass treated with a left partial nephrectomy. Pathologic examination demonstrated a clear cell RCC, WHO/ISUP grade 1, TNM stage pT1, with the parenchymal resection margin positive for tumor. Twenty-nine months following the partial nephrectomy, the patient was found to have a right testicular mass. Metastatic low-grade clear cell RCC to the testis was confirmed upon right radical orchiectomy. The metastatic tumor was two centimeters in size and confined to the testicular parenchyma. He underwent a completion left radical nephrectomy fourteen months after the orchiectomy, due to the local recurrence. With ongoing imaging surveillance, he was found to have several soft tissue masses in the left posterior chest wall, as well as masses in the pancreas. A two-centimeter enhancing mass was found in the upper pole of the right kidney 87 months postorchiectomy. The patient has been offered systemic therapy of VEGF inhibitor. He is alive with disease 93 months following the orchiectomy.

## 4. Gross Features

In four of five cases, the testicular tumors were unilateral; three tumors involved the right side and one tumor involved the left side. Three tumors were ipsilateral to the primary kidney tumor and one was contralateral. One case had bilateral testicular involvement. The tumors were well circumscribed, ranging from 2.0 cm to 5.7 cm in the greatest dimension. Four of the 5 cases were solitary masses. One case had multiple tumor nodules. On cut surface, the tumors were grayish-white to tan-yellow in color. The tumors in the case with bilateral testicular involvement had focal areas of hemorrhage and cystic spaces.

## 5. Microscopic Features

**Figure 1:**
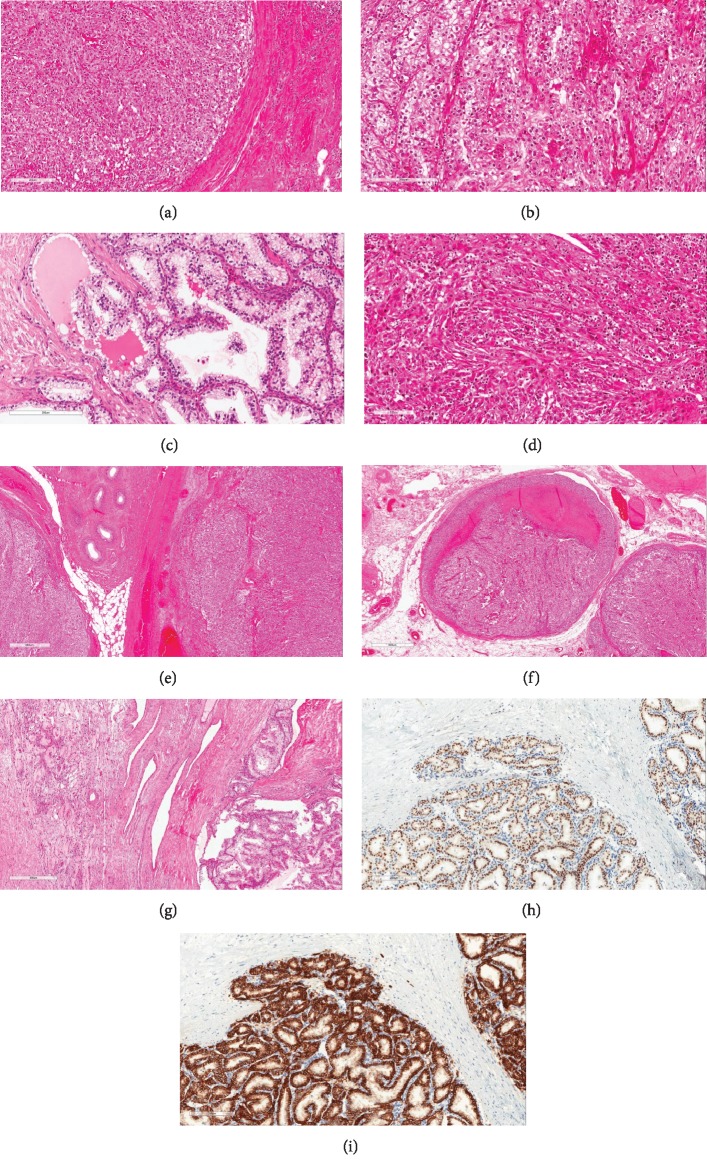
Microscopic examination of metastatic renal cell carcinoma in the testis; (a) the broad pushing border at the edge of the tumor; (b) the metastatic tumors showed mixed patterns of tubules, cysts, or well-vascularized nests; (c) low-grade area with cystic change, lined by clear cells and proteinaceous eosinophilic fluid; (d) small component of sarcomatoid dedifferentiation (WHO/ISUP grade 4); (e) tumor abutted the epididymis; (f) tumor present in the vessels in the hilar soft tissue; (g) residual testicular parenchyma showed decreased spermatogenesis, tubular atrophy, and fibrosis, with only interstitial Leydig cells remaining; (h) metastatic renal cell carcinoma was positive for PAX8; (i) metastatic renal cell carcinoma was positive for CAIX.

On a low-power view, all of the tumors involved the underlying testicular parenchyma and had broad pushing boarders (Figure 1(a)). The tumors had mixed patterns of hollow tubules, cysts, and alveoli or well-vascularized nests (Figure 1(b)). At high-power magnification, the polygonal tumor cells had a lightly eosinophilic to abundant clear cytoplasm. The cystic space contained macrophages and proteinaceous eosinophilic fluid (Figure 1(c)). The WHO/ISUP grades of the tumors were predominantly grade 2/4 (two cases) to grade 3/4 (two cases). In one case, the tumor had prominent vascularity, with a few areas of spindled carcinoma cells with increased hyperchromatism and pleomorphism, indicative of a small component of sarcomatoid dedifferentiation (WHO/ISUP grade 4) (Figure 1(d)). In two cases, there were direct extensions of the tumor into the rete testis, and in one of them, the tumor abutted the epididymis (Figure 1(e)). Lymphovascular invasion was noted in three cases (Figure 1(f)). Residual testicular parenchyma showed decreased spermatogenesis, tubular atrophy, and fibrosis, with unremarkable interstitial Leydig cells (Figure 1(g)). There was no evidence of intratubular germ cell neoplasia in any case. By immunohistochemistry, the metastatic RCC cells were positive for pan-keratin, PAX8 (Figure 1(h)), CD10, and CAIX (Figure 1(i)). They were variably to strongly positive for vimentin. The CK7 immunostain was negative in the tumor cells.

## 6. Discussion

Although RCC commonly results in metastases to various organs, the testicular spread of RCC is extremely rare. In the two largest autopsy series with renal cell carcinoma (1451 patients and 586 patients, respectively), none of them had testicular spread [21, 22]. The testes are regarded as a “tumor sanctuary,” as the relatively low temperature of the scrotum may provide unacceptable conditions for the establishment of metastatic tumor cells [3]. Additionally, the presence of the blood-testis barrier formed by Sertoli cells, which physiologically aims to protect spermatozoa, may also play an indirect role in the prevention of testicular metastasis [6]. Bandler et al. described the first case of RCC metastatic to the testis in 1946 [17]. Since then, there have been 34 reported cases of testicular spread of RCC [1–16]. The current report of testicular spread of RCC, with 5 cases, comprises one of the largest series to date and brings the total number of reported cases to 39. The major features of our presenting cases are summarized in Table 1.

Of the 39 reported cases, including five current cases, 17 involved left testes, 20 involved the right, and two cases demonstrated bilateral involvement. Our case 1 is the second reported case with bilateral involvement of RCC in the testis. The association of laterality between primary kidney tumors and the testis revealed 26 ipsilateral metastases, 11 contralateral metastases, and 2 bilateral metastases. The tendency for the metastasis to occur in the ipsilateral testis may be related to preferential routes of the spread between the kidney and the testis, including retrograde venous spread via the spermatic vein [7, 8, 12]. In the case reported by Moriyama, the primary kidney and the larger testicular metastasis had the same laterality, which supports the hypothesis of preferential ipsilateral metastasis [10]. However, in our case 1, the contralateral metastasis is larger than the ipsilateral one (5.7 cm versus 3.5 cm), leaving open the speculation that other venous or lymphatic conduits may be important in testicular metastasis of RCC. For example, Batson's venous complex, an avalvular venous network, may provide the necessary conduit between contralateral renal capsular veins and spermatic cord vessels, thus potentially explaining distant seeding to a contralateral testicle [4, 19]. Iatrogenic spread of cancer cells to testis may also contribute to the testicular metastasis of RCC, especially for the cases undergo partial nephrectomy with positive resection margins, while it is unlikely the reason for the patients with testicular metastasis as the initial presentation, such as the case 4 in this report.

The age of the previously reported cases ranged from 35 to 87 years old (median, 64 years), similar to the age range in our patients (53-81 years; median, 63 years). The time intervals between the identification of the primary kidney tumor and the discovery of testicular metastases in our cases ranged from 29 to 34 months following partial or radical nephrectomy in four of five cases, while in one case (case 4), the testicular mass was the initial presentation of the renal tumor. Follow-up information is available for all five patients in our series. The follow-up period following orchiectomy ranged from 14 to 93 months. Three patients died of the disease 17 to 42 months after orchiectomy. One patient (case 1) is alive with other metastatic lesions 14 months after orchiectomy. The last patient (case 5) had been free of disease 87 months after orchiectomy and is on targeted therapy for additional metastasis 93 months after orchiectomy. This case represents the longest follow-up and survival among all the reported cases to date. In this case, both the primary and metastatic tumor showed WHO/ISUP nuclear grade 1 to 2 features, although the primary resection had a positive parenchymal margin. The three patients who died of the disease all had WHO/ISUP nuclear grade 3 to 4 features, with sarcomatoid transformation in one of them. These findings support our understanding that prognosis correlates with the grade of the tumor.

The diagnosis of metastatic RCC should not be difficult once the pathologists are aware of this rare scenario. A previous history of RCC would be helpful, although it is not always known. Microscopically, a fine lacelike vascular network (Figure 1(b)) is typically seen in clear-cell RCC. Nonetheless, there are some primary testicular tumors morphologically resembling RCC. Sertoli or Sertoli-Leydig tumor can show solid nests and tubular patterns as in clear-cell RCC. However, they lack the intricate and delicate vascular network, instead thick-walled and sometimes hyalinized vessels may be prominent. Immunostains for inhibin are typically positive in Sertoli cell tumors [23, 24], while PAX8 immunostain is positive in RCC (Figure 1(h)). Classic seminoma, a common primary testicular tumor, may present with a prominence of clear cells as in metastatic RCC. However, seminoma patients are typically younger (mean age, 40 years) than those with metastatic RCC (mean age, 64 years). Furthermore, seminoma lacks the typical vasculature of clear cell RCC, instead, it usually demonstrates a conspicuous lymphocytic infiltrate, prominent stromal component, a background of germ cell neoplasia in situ, as well as the immunohistochemical positivity for PLAP [25, 26].

## 7. Conclusion

In conclusion, this study is one of the largest series of testicular metastasis of RCC. Our case 1 is the second reported case of simultaneous bilateral RCC metastatic to the testis. Our case 5 documents the longest follow-up and disease-free survival among all the reported cases to date. Metastatic tumors to the testis may be the initial presentation of RCC, and it is an important consideration that needs to be differentiated from primary germ cell tumor and sex cord stromal tumors of the testis.

## Figures and Tables

**Table 1:** Clinical characteristics of metastatic renal cell carcinoma to the testis.

Case no.	Age (year)	Type	Primary tumor side	Primary tumor grade	Primary tumor stage	LVI in primary tumor	Metastatic site(s)	Interval after primary (month)	Treatment for primary tumor	Treatment for metastatic tumor	Follow-up (month)	Outcome
1	53	Clear cell	Left	1	pT1	No	Bilateral testis	33	Ablation/partial nephrectomy	Radical orchiectomy	24	Alive with disease
2	81	Clear cell	Left	3	pT2a	No	Left testis, maxilla (14 months later)	34	Radical nephrectomy	Radical orchiectomy	15	Dead of disease
3	45	Clear cell	Right	4	pT2b	Yes	Right testis, bone (33 months later)	31	Radical nephrectomy	Radical orchiectomy, radiation	42	Dead of disease
4	63	Clear cell	Right	N/A	N/A	N/A	Right testis, brain, bone, lung	N/A	N/A	Radiation	17	Dead of disease
5	76	Clear cell	Left	1	pT1	No	Right testis	29	Partial/radical nephrectomy	Radical orchiectomy	93	Alive with disease
